# Dual Mutations in *MSMEG_0965* and *MSMEG_1380* Confer High-Level Resistance to Bortezomib and Linezolid by Both Reducing Drug Intake and Increasing Efflux in *Mycobacterium smegmatis*

**DOI:** 10.3390/ijms26083779

**Published:** 2025-04-17

**Authors:** Han Zhang, Cuiting Fang, Buhari Yusuf, Xiaoqing Zhu, Shuai Wang, H. M. Adnan Hameed, Yamin Gao, Tianyu Zhang

**Affiliations:** 1 School of Basic Medical Sciences, Division of Life Science and Medicine, University of Science and Technology of China, Hefei 230000, China; zhang_han@gibh.ac.cn; 2 State Key Laboratory of Respiratory Disease, Guangzhou Institutes of Biomedicine and Health, Chinese Academy of Sciences, Guangzhou 510000, Chinayusuf@gibh.ac.cn (B.Y.); wang_shuai@gibh.ac.cn (S.W.); adnan@gibh.ac.cn (H.M.A.H.); 3 Guangdong-HongKong-Macau Joint Laboratory of Infectious Respiratory Diseases, Guangzhou Institutes of Biomedicine and Health, Chinese Academy of Sciences, Guangzhou 510000, China; 4 China-New Zealand Belt and Road Joint Laboratory on Biomedicine and Health, Guangzhou Institutes of Biomedicine and Health, Chinese Academy of Sciences, Guangzhou 510000, China; 5 University of Chinese Academy of Sciences, Beijing 100000, China

**Keywords:** *Mycobacterium smegmatis*, bortezomib, linezolid, drug resistance mechanism

## Abstract

The emergence of multidrug-resistant and extensively drug-resistant *Mycobacterium tuberculosis* strains poses serious challenges to global tuberculosis control, highlighting the urgent need to elucidate the mechanisms underlying multidrug resistance. In this study, we screened for spontaneous bortezomib (BTZ)-resistant *Mycobacterium smegmatis* (Msm) mutants and identified a strain, Msm-R1-2, exhibiting 16- and 64-fold increases in minimum inhibitory concentrations (MICs) to BTZ and linezolid (LZD), respectively, compared to the parental strain. Whole-genome sequencing revealed resistance-associated mutations in two functionally distinct genes: *MSMEG_1380*, encoding a transcriptional regulator involved in efflux pump expression, and *MSMEG_0965*, encoding a porin protein. CRISPR-Cpf1-assisted gene knockout and editing experiments confirmed that single mutations in either *MSMEG_1380* or *MSMEG_0965* caused low-level resistance (4-fold MIC increase) to BTZ and LZD, while dual mutations conferred resistance levels comparable to Msm-R1-2, with 16- and 64-fold increases in MICs for BTZ and LZD, respectively. An ethidium bromide accumulation assay demonstrated that mutations in *MSMEG_0965* reduce cell wall permeability, contributing to multidrug resistance. Furthermore, quantitative real-time PCR showed that mutations in *MSMEG_1380* upregulate the *mmpS5*-*mmpL5* efflux system. Together, these dual mechanisms function synergistically: restricted drug entry combined with enhanced drug efflux confers robust multidrug resistance. These findings provide novel insights into the evolutionary mechanisms of resistance in mycobacteria.

## 1. Introduction

Tuberculosis (TB), caused by *Mycobacterium tuberculosis* (Mtb), remains a major global health challenge, especially due to the emergence of multidrug-resistant (MDR) and extensively drug-resistant (XDR) strains [1,2]. These resistant strains severely compromise current treatment regimens, underscoring the urgent need for novel therapeutic strategies [2,3]. Among the promising candidates, bortezomib (BTZ), a proteasome inhibitor originally approved for the treatment of multiple myeloma, has shown potent activity against Mtb [4]. Linezolid (LZD), an oxazolidinone antibiotic, is already a key component of TB treatment regimens [1,5]. However, the mechanisms underlying BTZ resistance remain poorly understood [6], and while LZD resistance has been primarily attributed to ribosomal mutations, non-ribosomal resistance mechanisms remain largely unexplored [7]. Thus, elucidating these resistance mechanisms is crucial for developing more effective therapeutic strategies.

Previous studies suggest that BTZ inhibits Mtb proteolysis by targeting ClpP1P2, a caseinolytic protease complex encoded by *clpP1* and *clpP2* [8,9,10], and may also target the Mtb proteasome complex PrcBA, encoded by *prcB* and *prcA* [11,12]. In contrast, LZD inhibits protein synthesis by binding to the 50S ribosomal subunit [13,14]. However, some resistant strains lack mutations in these established targets, implying the involvement of alternative resistance pathways, such as MmpL9, EmbB-EmbC, and GrcC1 [15,16,17,18]. Efflux pump systems, like the MmpS5-MmpL5 transporter, contribute to mycobacterial drug resistance by actively expelling antimicrobial compounds [19,20]. In *Mycobacterium smegmatis* (Msm), mutations in *MSMEG_1380*, encoding a TetR-family transcriptional regulator, upregulate efflux pump expression and confer resistance to multiple drugs including chrysomycin A [21,22]. Additionally, porins like MSMEG_0965 (MspA) are crucial for drug uptake by facilitating hydrophilic molecule transport across the mycobacterial cell wall [23,24]. Mutations in genes encoding porins can reduce membrane permeability, thereby diminishing the efficacy of antibiotics such as fluoroquinolones and potentially leading to treatment failure or prolonged therapy duration [25]. Although these resistance mechanisms have been individually characterized, their interactions in strains with multiple mutations remain unclear [26].

In this study, we used the nonpathogenic Msm mc^2^155 as a model organism [27] to investigate drug resistance mechanisms in mycobacteria. By screening spontaneous BTZ-resistant mutants and performing whole-genome sequencing (WGS), we identified multiple resistance-associated mutations, despite the absence of mutations in the known BTZ target, suggesting the involvement of alternative resistance mechanisms. Notably, among these mutants, we identified a strain exhibiting high-level resistance to both BTZ and LZD. CRISPR-Cpf1-assisted gene knockout, gene editing, overexpression, and complementation experiments revealed that *MSMEG_1380* and *MSMEG_0965* are key determinants of resistance. Single-gene mutations conferred low-level resistance (4-fold MIC increase), while double-gene mutations significantly enhanced resistance to BTZ, LZD, and other antibiotics (16- to 64-fold MIC increase). The ethidium bromide (EtBr) accumulation assay demonstrated that mutations in *MSMEG_0965* led to a significant reduction in cell wall permeability, resulting in lower intracellular drug concentrations. Furthermore, quantitative real-time PCR (qRT-PCR) showed that mutations in *MSMEG_1380* promote drug efflux through upregulation of the MmpS5-MmpL5 system. These results suggest that the synergy between reduced drug uptake (MSMEG_0965) and enhanced efflux (MmpS5-MmpL5) underscores a novel combinatorial resistance mechanism in mycobacteria, offering potential targets for countering MDR/XDR strains.

## 2. Results

### 2.1. High Frequency of Spontaneous Mutations Conferring BTZ Resistance in Msm

To investigate potential BTZ targets, we screened Msm mc^2^155 strains for spontaneous resistance to BTZ. First, we determined that the MIC of BTZ for Msm was 5 μg/mL. Msm strains were then plated on 7H10 agar plates containing increasing concentrations of BTZ (20 × MIC, 40 × MIC, and 60 × MIC) in four independent batches. From each batch, 10~20 single colonies were randomly selected, yielding a total of 57 resistant colonies. The MIC for each isolate was then determined to assess resistance levels. All isolates exhibited MIC ≥ 20 μg/mL (listed in Table S1), with a spontaneous mutation frequency ranging from 1.86 × 10^−7^ to 6.60 × 10^−7^. This frequency is higher than the reported spontaneous resistance mutation frequency of Mtb to LZD (2 × 10^−8^ to 5 × 10^−9^) [28], suggesting that Msm may exhibit increased mutability under BTZ exposure.

### 2.2. WGS of BTZ-Resistant Msm Strains Revealed a Variety of Mutations Across Different Genes

Sanger sequencing of *prcA*, *prcB*, *clpP1*, and *clpP2*, which encodes known BTZ targets, revealed no mutations in the resistant strains. To further explore potential resistance mechanisms, we randomly selected five resistant strains from the 57 isolates and the parent strain for WGS. WGS identified 35 unique mutations across various genes (Table S2). Notably, *MSMEG_3244* was the most frequently mutated gene (4/5 strains), followed by *MSMEG_5085* and *MSMEG_3987* (2/5 strains). Mutations in *MSMEG_1380* and *MSMEG_0965* were detected in only one strain. Among the 57 isolates analyzed through Sanger sequencing, mutations were detected in 5 strains for *MSMEG_3244*, 24 strains for *MSMEG_5085*, 10 strains for *MSMEG_3987*, and 2 strains each for *MSMEG_1380* and *MSMEG_0965*. Further validation is required to determine the association of these genes with BTZ resistance.

### 2.3. BTZ-Resistant Msm Strains Exhibited Cross-Resistance to Other Antibiotics

To determine whether the spontaneous drug-resistant mutants exhibited cross-resistance, we tested the susceptibility of WGS-analyzed strains to a range of antibiotics (Table 1). Most resistant strains showed no significant MIC changes (≤2-fold) in their sensitivity to antibiotics including levofloxacin (LEV), amikacin (AMK), and streptomycin (STR). However, some strains exhibited notable cross-resistance. For instance, Msm-R1-2 demonstrated high-level resistance to both BTZ and LZD, with MICs rising to 80 µg/mL and 128 µg/mL, respectively. Additionally, the MIC of clarithromycin (CLR) increased 4-fold. Similarly, Msm-R1-13 exhibited an 8-fold increase in MIC of CLR and a 4-fold increase in MIC of gentamicin (GEN), while Msm-R4-1 showed a 4-fold increase in MIC of ethambutol (EMB). These findings suggest that resistance mechanisms may involve shared pathways that regulate cross-resistance, warranting further investigation.

### 2.4. Single Knockout of MSMEG_1380 or MSMEG_0965 in Msm Resulted in Low-Level Resistance to BTZ and LZD

To investigate the genes associated with BTZ resistance in Msm, we used CRISPR-Cpf1-assisted recombineering technology [29] to knock out *MSMEG_3244*, *MSMEG_5085*, *MSMEG_3987*, *MSMEG_1380*, and *MSMEG_0965* genes. We then tested the MICs of BTZ against these knockout strains. The results showed that compared to Msm, Δ3244, Δ5085, and Δ3987 exhibited no significant changes in BTZ resistance, indicating that these genes are not involved in the resistance mechanism. In contrast, Δ1380 and Δ0965 showed a 4-fold increase in the MIC of BTZ compared to Msm, suggesting that *MSMEG_1380* and *MSMEG_0965* play crucial roles in Msm’s resistance to BTZ (Table 2).

Notably, Msm-R1-2 exhibited high-level resistance to both BTZ and LZD and carried mutations in *MSMEG_1380* and *MSMEG_0965* (Table 1 and Table S2). To determine their roles in resistance, we assessed the LZD susceptibility of Δ1380 and Δ0965. The results showed that, compared to wild-type Msm, both Δ1380 and Δ0965 exhibited a 4-fold increase in the MIC of LZD (Table 3). These findings suggest that *MSMEG_1380* and *MSMEG_0965* are key contributors to BTZ and LZD resistance in Msm. Based on the observed MIC shifts compared to the wild-type strain, we defined low-level resistance as a 4- to 8-fold increase in MIC (as observed in single mutants, Table 2 and Table 3), and high-level resistance as a ≥16-fold increase (as seen in Msm-R1-2, Table 1).

### 2.5. Single Knockout of MSMEG_1380 or MSMEG_0965 in Msm Affected the Sensitivity to Other Antibiotics

**Figure 1:**
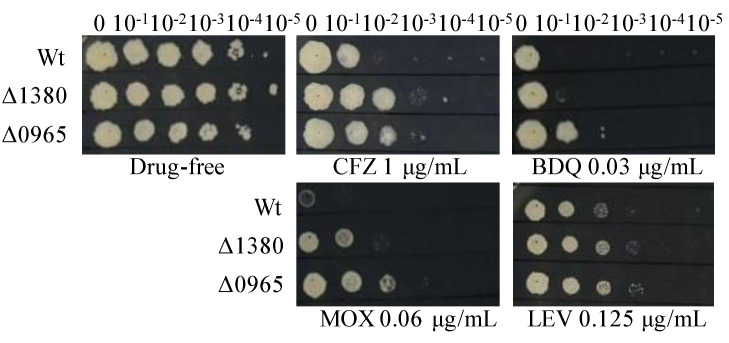
The sensitivity of Wt, Δ1380, and Δ0965 to various antibiotics. Wt, wild-type *Mycobacterium smegmatis* mc^2^155 strain (Msm); Δ1380, *MSMEG_1380* knockout Msm strain; Δ0965, *MSMEG_0965* knockout Msm strain. CFZ, clofazimine; BDQ, bedaquiline; MOX, moxifloxacin; LEV, levofloxacin. The experiment was repeated three times.

To investigate the antibiotic resistance profiles associated with *MSMEG_1380* and *MSMEG_0965*, we tested the susceptibility of Δ1380 and Δ0965 to a range of antibiotics. Both Δ1380 and Δ0965 exhibited resistance to clofazimine (CFZ), moxifloxacin (MOX), and LEV. Interestingly, bedaquiline (BDQ) resistance was observed only in Δ0965, indicating that *MSMEG_0965* plays a specific role in mediating BDQ resistance (Figure 1). In addition, both Δ1380 and Δ0965 displayed a 4-fold increase in MIC of vancomycin (VAN). Furthermore, Δ0965 also exhibited an 8-fold increase in MIC of CLR, a 4-fold increase in MIC of sulfadiazine (SDZ), and a 16-fold increase in MIC of sulfamethoxazole (SMX) (Table 4). In contrast, both knockout strains (Δ1380 and Δ0965) showed no significant differences in MICs for EMB, STR, GEN, and AMK compared to the wild-type Msm (Table S3). These findings suggest that the knockouts of *MSMEG_1380* and *MSMEG_0965* differentially affect antibiotic sensitivity, with gene-specific mechanisms such as BDQ resistance, which was mediated exclusively by *MSMEG_0965*.

### 2.6. Overexpression of MSMEG_1380 or MSMEG_0965 in Msm and Complementation of These Genes in Knockout Strains Affected the Drug Sensitivity to BTZ and LZD

**Figure 2:**
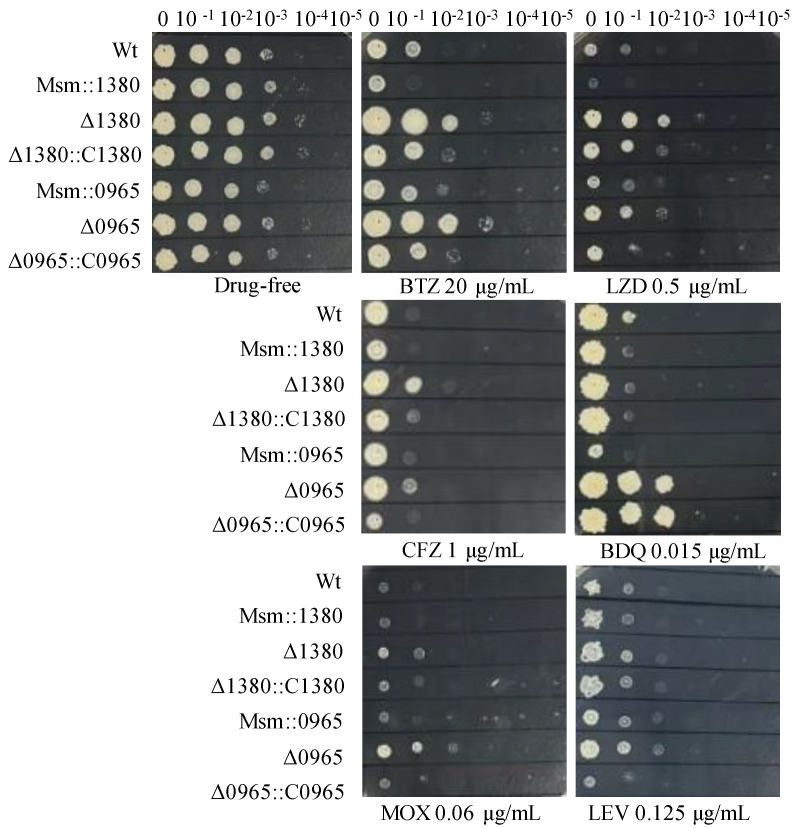
The sensitivity of recombinant strains to various antibiotics. Wt, wild-type Msm strain; Msm::1380, *MSMEG_1380* overexpression Msm strain; Δ1380, *MSMEG_1380* knockout Msm strain; Δ1380::C1380, *MSMEG_1380* complemented Δ1380 strain; Msm::0965, *MSMEG_0965* overexpression Msm strain; Δ0965, *MSMEG_0965* knockout Msm strain; Δ0965::C0965, *MSMEG_0965* complemented Δ0965 strain. BTZ, bortezomib; LZD, linezolid; CFZ, clofazimine; BDQ, bedaquiline; MOX, moxifloxacin; LEV, levofloxacin. The experiment was repeated three times.

To further investigate the functional contributions of *MSMEG_1380* and *MSMEG_0965* to drug susceptibility phenotypes, we generated Msm recombinant strains harboring overexpression constructs for these genes and performed genetic complementation in their respective knockout backgrounds. We expressed *MSMEG_1380* under the *hsp60* promoter and *MSMEG_0965* under its native promoter for overexpression and complementation studies. Drug susceptibility testing (DST) revealed that Msm::1380 and Msm::0965 strains displayed enhanced sensitivity to BTZ and LZD. The Δ1380::C1380 strain showed partially restored sensitivity to BTZ, LZD, and CFZ compared to Δ1380, while Δ0965::C0965 restored susceptibility to BTZ, LZD, MOX, and CFZ to the similar levels of wild-type Msm. Notably, BDQ susceptibility remained unaltered in the Δ0965::C0965 strain, suggesting a gene-specific interaction between *MSMEG_0965* and intrinsic BDQ resistance mechanisms (Figure 2). We also evaluated the susceptibility of these strains to other antibiotics, including VAN and CLR. The MICs of these antibiotics in the overexpression strains remained equivalent to those of wild-type Msm, and the MICs in the complementation strains showed no significant changes compared to their respective knockout strains (Table S4). These results suggest that overexpression of *MSMEG_1380* or *MSMEG_0965*, and complementation in the respective knockout strains affect specific drug sensitivity, but their underlying regulatory mechanisms differ, highlighting the complexity of the regulatory networks involved.

### 2.7. Roles of MSMEG_1380^12insC^ and MSMEG_0965^400insCC^ in Multidrug Resistance Were Confirmed by Gene Editing in Msm

**Figure 3:**
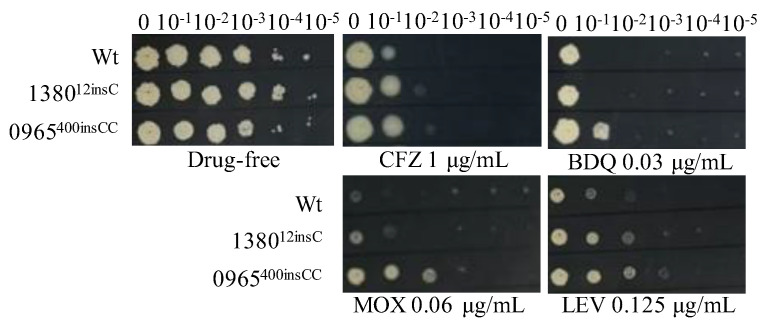
The sensitivity of single gene-edited strains to various antibiotics. Wt, wild-type Msm strain; 1380^12insC^, Msm strain with a 12insC insertion mutation in *MSMEG_1380*; 0965^400insCC^, Msm strain with a 400insCC insertion mutation in *MSMEG_0965*. CFZ, clofazimine; BDQ, bedaquiline; MOX, moxifloxacin; LEV, levofloxacin. The experiment was repeated three times.

To verify that single mutations in *MSMEG_1380* or *MSMEG_0965* confer resistance to multiple drugs, we introduced these mutations using CRISPR-Cpf1-assisted recombineering technology [29]. The drug susceptibility profiles of the single gene-edited strains were consistent with those of Δ1380 and Δ0965 (Figure 1 and Figure 3, Table 4 and Table 5). These results indicated that single knockout or mutation of *MSMEG_1380* or *MSMEG_0965* in Msm conferred only low-level resistance to BTZ and LZD rather than the high-level resistance observed in the Msm-R1-2 strain. These findings confirm the important roles of *MSMEG_1380* and *MSMEG_0965* in conferring drug resistance in Msm. However, the precise mechanisms underlying the high-level resistance to BTZ and LZD in the Msm-R1-2 strain require further study.

### 2.8. Dual Mutations of MSMEG_1380 and MSMEG_0965 in Msm Exhibited High-Level Resistance to BTZ and LZD

**Figure 4:**
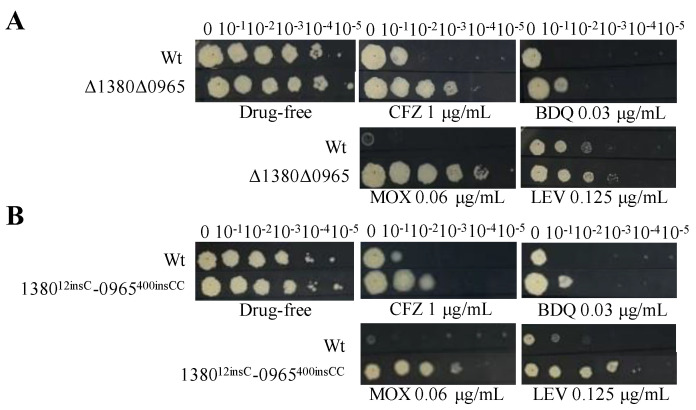
Dual mutations of *MSMEG_1380* and *MSMEG_0965* affect Msm sensitivity to various antibiotics. (**A**) The sensitivity of Wt and Δ1380Δ0965 to various antibiotics. (**B**) The sensitivity of Wt and 1380^12insC^-0965^400insCC^ to various antibiotics. Wt, wild-type Msm strain; Δ1380Δ0965, *MSMEG_1380* and *MSMEG_0965* double-knockout Msm strain; 1380^12insC^-0965^400insCC^, Msm strain with a 12insC insertion in *MSMEG_1380* and a 400insCC insertion in *MSMEG_0965*. CFZ, clofazimine; BDQ, bedaquiline; MOX, moxifloxacin; LEV, levofloxacin. The experiment was repeated three times.

Based on the above results, we hypothesize that the dual mutations in *MSMEG_1380* and *MSMEG_0965* are the primary cause of high-level resistance in Msm [30,31]. To validate this hypothesis, we used CRISPR-Cpf1-assisted recombineering technology [29] to perform the dual knockouts and gene editing of these two genes in Msm. DST revealed that the MICs of BTZ and LZD against the dual mutation strains (Δ1380Δ0965 and 1380^12insC^-0965^400insCC^) were 16-fold and 64-fold higher, respectively, than those to Msm (Table 6), which was consistent with the MIC values observed in Msm-R1-2 (Table 1). Compared to the single-gene mutant strains, the dual mutation strains also exhibited greater resistance to MOX and LEV (Figure 1, Figure 3 and Figure 4), and increased resistance to VAN (Table 4, Table 5 and Table 6). The resistance levels of the double mutation strains to CLR, SDZ, and SMX were comparable to those of Δ0965 and 0965^400insCC^ (Table 4, Table 5 and Table 6). Interestingly, the complementation of *MSMEG_1380* and *MSMEG_0965* in the double-knockout strains did not alter the MICs of multiple drugs (Table 6). Based on the MIC results of the dual knockout and dual gene-edited strains, we propose that the dual mutations of *MSMEG_1380* and *MSMEG_0965* in Msm are the main reason for Msm’s high-level resistance to BTZ and LZD, and they also influence the sensitivity to other antibiotics.

### 2.9. The Knockout and Mutation of MSMEG_0965 Reduced Cell Wall Permeability

**Figure 5:**
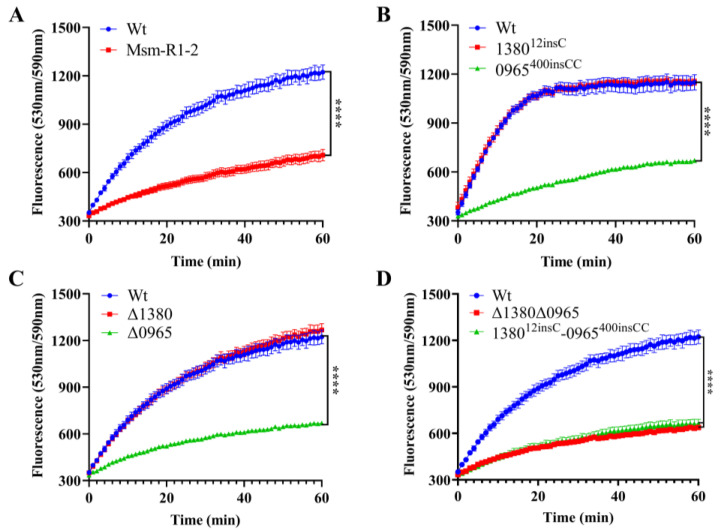
*MSMEG_0965* affects cell wall permeability in Msm. (**A**) EtBr accumulation in Wt and Msm-R1-2. (**B**) EtBr accumulation in single gene-edited strains. (**C**) EtBr accumulation in single knockout strains. (**D**) EtBr accumulation in double-knockout and double gene-edited strains. ^****^, *p* < 0.0001. Data are presented as mean ± standard deviation, *n* = 3. Wt, wild-type Msm strain; Msm-R1-2, BTZ-resistant strain; Δ1380, *MSMEG_1380* knockout Msm strain; Δ0965, *MSMEG_0965* knockout Msm strain; 1380^12insC^, Msm strain with a 12insC insertion mutation in *MSMEG_1380*; 0965^400insCC^, Msm strain with a 400insCC insertion mutation in *MSMEG_0965*; Δ1380Δ0965, *MSMEG_1380,* and *MSMEG_0965* double-knockout Msm strain; 1380^12insC^-0965^400insCC^, Msm strain with a 12insC insertion in *MSMEG_1380* and a 400insCC insertion in *MSMEG_0965*.

To assess the impact of *MSMEG_0965* on cell wall integrity [32,33], we performed the EtBr accumulation assay in Msm-R1-2, knockout and gene-edited Msm strains [34]. The results showed that Msm-R1-2, Δ0965 and 0965^400insCC^ strains exhibited significantly reduced cell wall permeability, while the permeability of Δ1380 and 1380^12insC^ remained unchanged. Similarly, the permeability of Δ1380Δ0965 and 1380^12insC^-0965^400insCC^ was also significantly decreased (Figure 5). These results indicate that knockout and mutation of *MSMEG_0965* reduce cell wall permeability, thereby contributing to drug resistance, while *MSMEG_1380* does not appear to affect cell wall permeability.

### 2.10. The Knockout and Mutation of MSMEG_1380 Upregulate the Expression of the mmpS5-mmpL5 Efflux System

**Figure 6:**
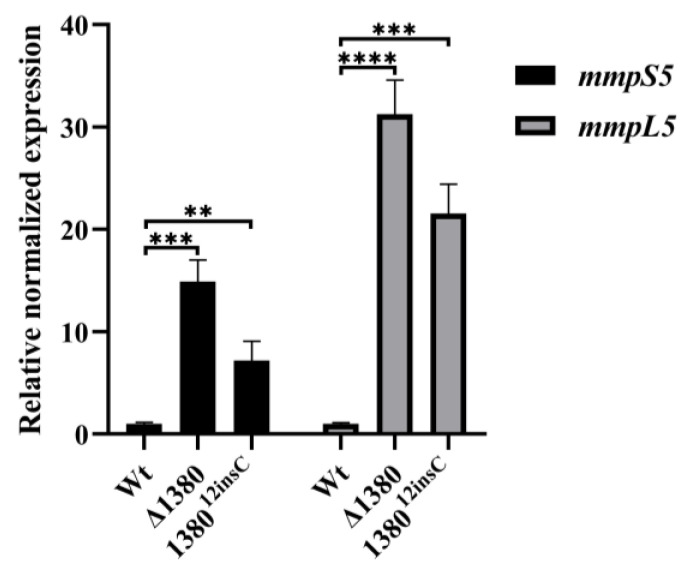
*MSMEG_1380* affects the relative expression levels of the *mmpS5-mmpL5* operon genes in Msm. **, *p* < 0.01; ***, *p* < 0.001; ****, *p* < 0.0001. Wt, wild-type Msm strain; Δ1380, *MSMEG_1380* knockout Msm strain; 1380^12insC^, Msm strain with a 12insC insertion mutation in *MSMEG_1380*.

Given the observed resistance phenotype in *MSMEG_1380* mutants, we examined the expression levels of *mmpS5* and *mmpL5* in the mutant strains [22]. The results demonstrated that compared to the wild-type Msm, *mmpS5* expression increased 14.89-fold in Δ1380 and 7.16-fold in 1380^12insC^, while *mmpL5* expression increased 31.24-fold and 21.53-fold, respectively (Figure 6). These findings confirm that mutations in *MSMEG_1380* significantly enhance the *mmpS5*-*mmpL5* operon expression, thereby increasing drug efflux capacity.

## 3. Discussion

The emergence of MDR and XDR Mtb strains presents a critical barrier to global TB control, emphasizing the urgent need for new therapeutic strategies [1,35]. Our study demonstrates that dual mutations in *MSMEG_1380* and *MSMEG_0965* synergistically confer high-level resistance to BTZ and LZD through a coordinated modulation of efflux and uptake, revealing a previously unrecognized combinatorial mechanism in mycobacteria.

To dissect the genetic basis of resistance, we conducted a genomic analysis that validated the specificity of this mechanism. In the obtained BTZ-resistant Msm strains, neither Sanger sequencing nor WGS detected no mutations in known BTZ target genes (*prcA*, *prcB*, *clpP1*, and *clpP2*) [4,10] or in genes associated with LZD resistance (*rplC*, *rrl*, *mmpL9*, *embB-embC*, and *grcC1*) [15,16,17]. While we identified an *rpoB* mutation (associated with RIF resistance) in Msm-R1-2 [36], its phenotypic impact was minimal: only a 2-fold increase in RIF MIC, compared to 16- and 64-fold increases for BTZ and LZD, respectively. Moreover, the MIC shifts observed in *MSMEG_0965* single mutants (Table S3) were consistent with this pattern. These results suggest that classical resistance pathways are not major contributors to the observed resistance.

Further phenotypic characterization of single-gene knockout strains revealed distinct resistance patterns, with Δ0965 but not Δ1380 conferring resistance to BDQ, CLR, SDZ, and SMX (Figure 1, Table 3, Table 4 and Table 5). The specific dependence of BDQ resistance on the MspA porin encoded by *MSMEG_0965* [23,37,38,39] suggests this porin mediates in BDQ uptake. Meanwhile, the absence of CLR resistance in Δ1380 indicates that CLR efflux likely occurs through alternative systems such as ABC transporters rather than the MSMEG_1380-mediated MmpS5-MmpL5 system [40]. The particular sensitivity of *MSMEG_0965* mutations strains to SMX and SDZ can be explained by their hydrophilic nature (logP = 0.79 and 0.25, respectively, obtained from https://go.drugbank.com/ (accessed on 31 March 2025), which makes them more dependent on porin-mediated uptake. In contrast, resistance to both BTZ and LZD appears to be dually influenced by both *MSMEG_1380* and *MSMEG_0965*. In addition, complementation and overexpression experiments showed that *MSMEG_1380* and *MSMEG_0965* influence drug sensitivity to BTZ, LZD, and some other antibiotics, but their effects on drugs like LEV, VAN, and CLR remained unchanged. This aligns with studies showing that target overexpression or complementation can have drug-specific effects due to mechanistic differences [18,41].

**Figure 7:**
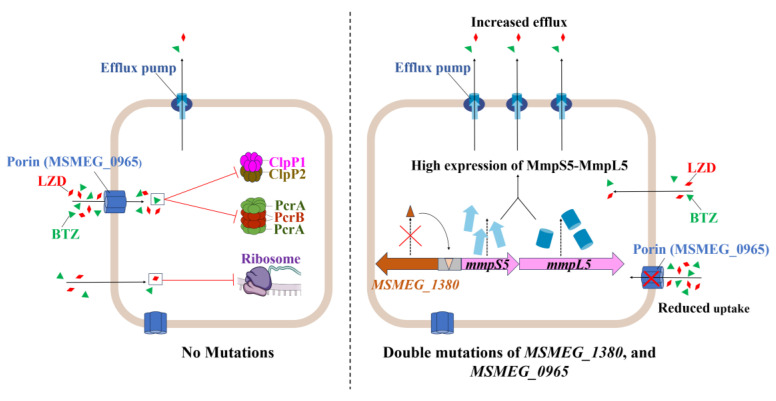
Schematic representation of the mechanism underlying high-level resistance to BTZ and LZD in Msm harboring mutations in *MSMEG_1380* and *MSMEG_0965*.

Building on these findings, the synergistic resistance observed in the double mutant likely results from the combined disruption of uptake and efflux processes, as illustrated in Figure 7 [42,43,44]. To assess the broader relevance of this mechanism, we examined homologs in pathogenic mycobacteria. For example, the *Rv0678* gene (the *MSMEG_1380* homolog in Mtb) promotes efflux-mediated resistance to BDQ and CFZ [45,46]. Similarly, mutations in *MAB_4384* (the homolog of *MSMEG_1380* in *Mycobacterium abscessus*) are linked to LZD resistance [47]. Although Mtb lacks a homolog of MSMEG_0965 (MspA) [48], expressing MspA in Mtb enhances drug susceptibility [37], suggesting the presence of compensatory uptake mechanisms, such as the aquaporin Rv1698 [49]. Thus, despite molecular divergence, coordinated efflux–uptake regulation appears to be a conserved resistance strategy across mycobacteria.

However, the interaction between *MSMEG_1380* and *MSMEG_0965* remains unclear. Their synergistic phenotype suggests potential coordination at transcriptional or post-translational levels. Such coordination between efflux and uptake is an evolutionarily conserved strategy for antibiotic resistance, as observed in *Pseudomonas aeruginosa*, where the MexAB-OprM efflux system cooperates with outer membrane permeability barriers to enhance drug resistance [50]. Based on this, we propose that reduced drug uptake due to *MSMEG_0965* mutations may lower the intracellular antibiotic burden, thereby enhancing the efficiency of MSMEG_1380-mediated efflux. Conversely, increased efflux activity may impose selective pressure favoring porin mutations, further restricting drug entry. This interplay could establish a feedback loop, ultimately contributing to high-level resistance in dual mutants.

Despite these advances, this study has limitations. First, while our findings in Msm provide mechanistic insights, direct validation in clinical Mtb isolates remains essential to establish the translational relevance of this dual resistance model. Second, while the phenotypic effects of *MSMEG_1380* and *MSMEG_0965* mutations are well characterized, their precise molecular mechanisms are not yet fully understood. Functional studies, including protein structure–function analyses and investigations of regulatory pathways, are essential to further elucidate their roles. Finally, our study focused primarily on these two key determinants, leaving other potential resistance pathways and mutations identified in our screening (Table S2) unexplored, which warrants future investigation to obtain a more comprehensive understanding of mycobacterial drug resistance.

Future studies should focus on validating these findings in Mtb clinical isolates to establish clinical relevance, such as through heterologous expression of *MSMEG_0965* or knockout of *Rv0678* in Mtb, particularly to assess their roles in BTZ and LZD resistance. Further elucidation of *MSMEG_1380* and *MSMEG_0965* mechanisms is essential. Investigating their potential transcriptional co-regulation via RNA-seq or ChIP-seq and analyzing protein–protein interactions through pull-down assays or bacterial two-hybrid systems could clarify their regulatory relationships and physical associations. Additionally, a systematic analysis of other resistance-associated pathways identified in our screening data (Table S2) will help establish a more comprehensive model of mycobacterial drug resistance. From a therapeutic perspective, targeting the MmpS5-MmpL5 efflux system or restoring porin-mediated drug uptake may offer promising strategies to counteract resistance. Finally, systematic investigation of BTZ resistance mechanisms will be crucial for gaining deeper insights and providing the basis for the development of next-generation anti-TB therapies.

## 4. Materials and Methods

### 4.1. Strains, Plasmids, and Culture Conditions

All mycobacterial strains were cultured at 37 °C in 7H9 liquid medium (BD Difco, Sparks, MD, USA) supplemented with 0.05% Tween 80 (Amresco/VWR, Solon, OH, USA), 0.2% glycerol (Macklin, Shanghai, China), and 10% OADC (BD Difco, Sparks, MD, USA), or on 7H10/7H11 solid medium (BD Difco, Sparks, MD, USA). Antibiotics were added to the media as required: 50 μg/mL kanamycin (KAN, Solarbio, Beijing, China) and 30 μg/mL zeocin (ZEO, InvivoGen, Toulouse, France). For induced gene expression, 200 ng/mL anhydrotetracycline (aTc, Solarbio, Beijing, China) was added. All *Escherichia coli* strains were cultured in LB medium (prepared in-house using NaCl, agar, tryptone and yeast extract from Maikesi Biological Technology Co., Ltd., Shenzhen, China), supplemented with 50 μg/mL KAN, 30 μg/mL ZEO, and 100 μg/mL ampicillin as required.

### 4.2. Screening for Spontaneous BTZ-Resistant Msm Strains

Msm cultures at an OD_600_ of 1.0 were plated (500 μL) on 7H10 medium containing 100, 200, or 300 μg/mL BTZ (AbMole, Wuhan, China), with two replicates per concentration. Serial 10-fold dilutions were then plated on BTZ-free 7H10 agar to calculate colony-forming units (CFU). The mutation frequency was determined by counting CFU and the number of resistant colonies. Resistant strains were analyzed for mutations in *prcA*, *prcB*, *clpP1*, and *clpP2* by Sanger sequencing using primers listed in Table S5. Genomic DNA from the parent strain and seven BTZ-resistant strains (which did not harbor mutations in *prcA*, *prcB*, *clpP1*, or *clpP2*) was sequenced by Shanghai Jingnuo Biotechnology Co., Ltd. (Shanghai, China). Mutations in resistant strains were identified by comparing their genomic profiles with those of the parent strain.

### 4.3. Construction of Knockout Strains

Gene knockout was performed using CRISPR-Cpf1-assisted recombineering technology [29]. First, the pJV53-Cpf1 plasmid was electroporated into Msm to obtain Msm::pJV53-cpf1 and then induced competent cells were prepared. The crRNA sequences were designed using the website (https://chopchop.cbu.uib.no/ (accessed on 27 April 2024), synthesized by Sangon Biotech (Shanghai) Co., Ltd. (Guangzhou, China) (Table S6), and ligated into the linearized pCR-Zeo vector after annealing. We amplified ~800 bp homology arms (containing 15–36 bp of the target gene) and inserted them into pBlueSK. The assembled ~1600 bp fragment was amplified by PCR to generate the repair template. The pCR-Zeo plasmid containing the crRNA sequence and the double-stranded template were then electroporated into the Msm::pJV53-cpf1 inducible competent cells. The cells were plated on 7H11 agar containing 50 μg/mL KAN, 30 μg/mL ZEO, and 200 ng/mL aTc and incubated at 30 °C. Positive colonies were identified by PCR and Sanger sequencing.

### 4.4. Construction of Overexpression and Complementation Strains

The target genes were PCR-amplified from Msm and cloned into the linearized pRH2502 plasmid under the control of either the exogenous *hsp60* promoter or the native promoter of the respective genes. Primers used in this study are listed in Table S5. The recombinant plasmids were electroporated into either wild-type Msm (for overexpression strains) or the corresponding knockout strains (for complementation strains). Transformants were plated on 7H10 agar containing 50 μg/mL KAN, and positive colonies were confirmed by PCR.

### 4.5. Construction of Gene Editing Strains

CRISPR-Cpf1-assisted recombineering [29] was used for gene editing. CrRNAs targeting the mutation sites were designed using the CHOPCHOP web tool (Table S6) and subsequently inserted into the linearized pCR-Zeo vector. The 500 bp flanking regions (~1000 bp total) of the mutation site were amplified from the resistant strains and used as repair templates. The crRNA-containing pCR-Zeo plasmid and repair templates were co-electroporated into Msm::pJV53-Cpf1 inducible competent cells. Transformants were plated on 7H11 agar containing 50 μg/mL KAN, 30 μg/mL ZEO, and 200 ng/mL aTc and incubated at 30 °C. Positive colonies were verified by PCR and Sanger sequencing.

### 4.6. DST

In this study, all drugs were prepared in solutions with DMSO (Xilong Scientific Co., Ltd., Shantou, China) or water at a final concentration of 10 mg/mL, except for CFZ (Meilunbio, Dalian, China), which was prepared at a final concentration of 5 mg/mL. Two methods were used to test the drug susceptibility of the strains. Knockout, overexpression, and complementation strains were grown in 7H9 medium at 37 °C to the logarithmic phase for both methods. All MIC assays were performed in three independent experiments.

According to CLSI guidelines [51], we performed the microplate broth dilution assay. Bacterial cultures were adjusted to an OD_600_ of 0.5 and subsequently diluted 1:1000. The MICs were determined using the broth microdilution method in 96-well plates. Cultures were incubated at 37 °C for three days, and the MIC was defined as the lowest concentration of antibiotic that visibly inhibited bacterial growth [17].

For the solid plate assay, cultures were adjusted to an OD_600_ of 0.9. Bacterial suspensions were serially diluted in 10-fold increments to obtain six different dilutions, ranging from the undiluted suspension to a 10^−5^ dilution. A 1 µL aliquot of each dilution was spotted onto solid media containing different concentrations of drugs, with 7H10 solid media without antibiotics serving as the control. The plates were incubated at 37 °C for three days, and colony growth was assessed visually and photographed.

### 4.7. Cell Wall Permeability Assay

The EtBr accumulation assay was performed as described previously [34]. We prepared the 4 μg/mL EtBr (Macklin, Shanghai, China) working solution in PBS (GENOM, Hangzhou, China) containing 0.08% glucose (Macklin, Shanghai, China) and 0.05% Tween 80. Cultures were grown to an OD_600_ of 0.6–0.8, centrifuged, and resuspended in PBS with 0.05% Tween 80 to an OD_600_ of 0.5. For each strain, 100 μL of the cell suspension was added to a white 96-well plate (3 experimental wells, 3 no-reagent controls, and 3 no-cell controls). Fluorescence was measured using a multimode microplate reader (excitation: 530 nm, emission: 590 nm) at 1 min intervals over 60 min. Background fluorescence was subtracted, and data were analyzed using GraphPad Prism 8.0.2. Results are presented as the mean ± SD, with *n* = 3 for each group. Statistical significance was assessed using an independent *t*-test, with *p* < 0.05 considered significant.

### 4.8. RNA Isolation and qRT-PCR

Total RNA extraction and qRT-PCR analysis were performed following standardized protocols [22]. Briefly, bacterial strains were cultured to an OD_600_ of 0.8–1.0 before harvesting the cells. Cell disruption was achieved through liquid nitrogen grinding, followed by total RNA extraction using the HiPure Bacterial RNA Kit (Magen, Guangzhou, China). For cDNA synthesis, 2 μg of total RNA was reverse transcribed using HiScript Q RT SuperMix for qPCR (Vazyme, Nanjing, China). The primers used for qRT-PCR are listed in Table S5. qRT-PCR was performed on a CFX96 Touch Real-Time PCR Detection System (Bio-Rad, Hercules, CA, USA) using 10-fold diluted cDNA as a template and Taq Pro Universal SYBR qPCR Master Mix (Vazyme, Nanjing, China). The gene *sigA* served as a reference, and relative gene expression levels were calculated using the 2^(−ΔΔCT)^ method. All data were analyzed using GraphPad Prism 8.0.2, with three biological replicates included for each sample to ensure experimental reliability. Statistical significance was determined by Student’s *t*-test, with *p* < 0.05 considered statistically significant.

## Figures and Tables

**Table 1:** Minimum inhibitory concentrations (MICs) of various antibiotics against different spontaneous *Mycobacterium smegmatis* mc^2^155 strains (Msm).

Strains	MICs (μg/mL)/Fold Change							
	BTZ	LZD	CLR	EMB	GEN	LEV	AMK	STR
Wt	5/1	2/1	1/1	1/1	2/1	0.25/1	0.5/1	0.5/1
Msm-R1-2 ^#^	80/16	128/64	4/4	2/2	2/1	0.25/1	0.5/1	0.25/0.5
Msm-R1-13 ^#^	>80/>16	4/2	8/8	1/1	8/4	0.25/1	0.5/1	0.25/0.5
Msm-R3-2 ^#^	80/16	4/2	2/2	2/2	4/2	0.25/1	0.5/1	1/2
Msm-R4-1 ^#^	80/16	2/1	2/2	4/4	1/0.5	0.5/2	0.5/1	0.5/1
Msm-R4-7 ^#^	80/16	2/1	1/1	1/1	1/0.5	0.5/2	1/2	0.5/1

Wt, wild-type Msm strain; ^#^, BTZ-resistant Msm strains. BTZ, bortezomib; LZD, linezolid; CLR, clarithromycin; EMB, ethambutol; GEN, gentamicin; LEV, levofloxacin; AMK, amikacin; STR, streptomycin. The experiment was performed in triplicate and repeated three times.

**Table 2:** MICs of BTZ against different gene knockout Msm strains.

Strains	MIC (μg/mL)/Fold Change
Wt	5/1
Δ3244	5/1
Δ5085	5/1
Δ3987	5/1
Δ1380	20/4
Δ0965	20/4

Wt, wild-type Msm strain; Δ3244, *MSMEG_3244* knockout Msm strain; Δ5085, *MSMEG_5085* knockout Msm strain; Δ3987, *MSMEG_3987* knockout Msm strain; Δ1380, *MSMEG_1380* knockout Msm strain; Δ0965, *MSMEG_0965* knockout Msm strain. BTZ, bortezomib. The experiment was performed in triplicate and repeated three times.

**Table 3:** MICs of LZD against different gene knockout Msm strains.

Strains	MIC (μg/mL)/Fold Change
Wt	2/1
Δ1380	8/4
Δ0965	8/4

Wt, wild-type Msm strain; Δ1380, *MSMEG_1380* knockout Msm strain; Δ0965, *MSMEG_0965* knockout Msm strain. LZD, linezolid. The experiment was performed in triplicate and repeated three times.

**Table 4:** MICs of multiple drugs against different gene knockout Msm strains.

Strains	MICs (μg/mL)/Fold Change			
	VAN	CLR	SDZ	SMX
Wt	8/1	2/1	2/1	1/1
Δ1380	32/4	2/1	2/1	1/1
Δ0965	32/4	16/16	8/4	16/16

Wt, wild-type Msm strain; Δ1380, *MSMEG_1380* knockout Msm strain; Δ0965, *MSMEG_0965* knockout Msm strain. VAN, vancomycin; CLR, clarithromycin; SDZ, sulfadiazine; SMX, sulfamethoxazole. The experiment was performed in triplicate and repeated three times.

**Table 5:** MICs of multiple drugs against single gene-edited strains.

Strains	MICs (μg/mL)/Fold Change					
	BTZ	LZD	VAN	CLR	SDZ	SMX
Wt	5/1	2/1	8/1	2/1	2/1	1/1
1380^12insC^	20/4	8/4	32/4	2/1	2/1	1/1
0965^400insCC^	20/4	8/4	32/4	16/8	8/4	16/16

Wt, wild-type Msm strain; 1380^12insC^, Msm strain with a 12insC insertion mutation in *MSMEG_1380*; 0965^400insCC^, Msm strain with a 400insCC insertion mutation in *MSMEG_0965*. BTZ, bortezomib; LZD, linezolid; VAN, vancomycin; CLR, clarithromycin; SDZ, sulfadiazine; SMX, sulfamethoxazole. The experiment was performed in triplicate and repeated three times.

**Table 6:** MICs of multiple drugs against different strains.

Strains	MICs (μg/mL)/Fold Change					
	BTZ	LZD	VAN	CLR	SDZ	SMX
Wt	5/1	2/1	8/1	2/1	2/1	1/1
Δ1380Δ0965	80/16	128/64	128/16	16/8	16/8	16/16
1380^12insC^-0965^400insCC^	80/16	128/64	128/16	16/8	16/8	16/16
Δ1380Δ0965::C1380C0965	80/16	128/64	128/16	16/8	16/8	16/16

Wt, wild-type Msm strain; Δ1380Δ0965, *MSMEG_1380* and *MSMEG_0965* double-knockout Msm strain; 1380^12insC^-0965^400insCC^, Msm strain with a 12insC insertion in *MSMEG_1380* and a 400insCC insertion in *MSMEG_0965*; Δ1380Δ0965::C1380C0965, *MSMEG_1380* and *MSMEG_0965* double complemented Δ1380Δ0965 strain. BTZ, bortezomib; LZD, linezolid; VAN, vancomycin; CLR, clarithromycin; SDZ, sulfadiazine; SMX, sulfamethoxazole. The experiment was performed in triplicate and repeated three times.

## Data Availability

All relevant data are included within the paper and its Supplementary Materials.

## Supplementary Materials

The following supporting information can be downloaded at https://www.mdpi.com/article/10.3390/ijms26083779/s1.
