# Supplementary material for: Dual Mutations in MSMEG_0965 and MSMEG_1380 Confer High-Level Resistance to Bortezomib and Linezolid by Both Reducing Drug Intake and Increasing Efflux in Mycobacterium smegmatis
Source: Int J Mol Sci. 2025 Apr 17;26(8):3779. doi: 10.3390/ijms26083779 (PMC12028319; doi:10.3390/ijms26083779)
Supplement: Supplementary file 1 [file ijms-26-03779-s001.zip › ijms-3526049-supplementary.pdf]

Supplementary Materials

for

Dual Mutations in *MSMEG_0965* and *MSMEG_1380* Confer High-Level Resistance to Bortezomib and Linezolid by both Reducing Drug Intake and Increasing Efflux in *Mycobacterium smegmatis*

Han Zhang [1,2,3,4], Cuiting Fang [2,3,4], Buhari Yusuf [2,3,4,5], Xiaoqing Zhu [4], Shuai Wang [2,3,4,5], H.M. Adnan Hameed [2,3,4,5], Yamin Gao [2,3,4,5,*] and Tianyu Zhang [1,2,3,4,5,*]

[1]School of Basic Medical Sciences, Division of Life Science and Medicine, University of Science and Technology of China, Hefei 230000, China; zhang_han@gibh.ac.cn

[2]State Key Laboratory of Respiratory Disease, Guangzhou Institutes of Biomedicine and Health, Chinese Academy of Sciences, Guangzhou 510000, China; yusuf@gibh.ac.cn (B.Y.); wang_shuai@gibh.ac.cn (S.W.); adnan@gibh.ac.cn (H.M.A.H.)

[3]Guangdong-HongKong-Macau Joint Laboratory of Infectious Respiratory Diseases, Guangzhou Institutes of Biomedicine and Health, Chinese Academy of Sciences, Guangzhou 510000, China

[4]China China-New Zealand Belt and Road Joint Laboratory on Biomedicine and Health, Guangzhou Institutes of Biomedicine and Health, Chinese Academy of Sciences, Guangzhou 510000, China

[5]University of Chinese Academy of Sciences, Beijing 100000, China

[*]Correspondence: gao_yamin@gibh.ac.cn (Y.G.); zhang_tianyu@gibh.ac.cn (T.Z.)

Table S1. MICs of BTZ against BTZ-resistant Msm strains.

Strains	MIC (μg/mL)/fold change	Strains	MIC (μg/mL)/fold change
Wt	5/1	Msm-R2-11	>80/>16
Msm-R1-1	40/8	Msm-R2-12	>80/>16
Msm-R1-2	80/16	Msm-R2-13	>80/>16
Msm-R1-3	40/8	Msm-R2-14	80/16
Msm-R1-4	40/8	Msm-R2-15	80/16
Msm-R1-5	80/16	Msm-R3-1	80/16
Msm-R1-6	80/16	Msm-R3-2	80/16
Msm-R1-7	40/8	Msm-R3-3	80/16
Msm-R1-8	40/8	Msm-R3-4	40/8
Msm-R1-9	80/16	Msm-R3-5	>80/>16
Msm-R1-10	40/8	Msm-R3-6	>80/>16
Msm-R1-11	>80/>16	Msm-R3-7	>80/>16
Msm-R1-12	>80/>16	Msm-R3-8	>80/>16
Msm-R1-13	>80/>16	Msm-R3-9	80/16
Msm-R1-14	80/16	Msm-R4-1	80/16
Msm-R1-15	40/8	Msm-R4-2	40/8
Msm-R1-16	>80/>16	Msm-R4-3	40/8
Msm-R1-17	80/16	Msm-R4-4	40/8
Msm-R1-18	80/16	Msm-R4-5	80/16
Msm-R2-1	>80/>16	Msm-R4-6	80/16
Msm-R2-2	>80/>16	Msm-R4-7	80/16
Msm-R2-3	>80/>16	Msm-R4-8	80/16
Msm-R2-4	80/16	Msm-R4-9	40/8
Msm-R2-5	80/16	Msm-R4-10	>80/>16
Msm-R2-6	>80/>16	Msm-R4-11	>80/>16
Msm-R2-7	>80/>16	Msm-R4-12	>80/>16
Msm-R2-8	>80/>16	Msm-R4-13	>80/>16
Msm-R2-9	80/16	Msm-R4-14	>80/>16
Msm-R2-10	>80/>16	Msm-R4-15	20/4

Wt, wide type Msm strain. All other strains were BTZ-resistant Msm strains. BTZ, bortezomib. The experiment was repeated three times.

Table S2. Mutations identified from WGS of BTZ-resistant Msm strains.

Strains	Gene	Mutations (base/ amino acids)	Product
Msm-R1-2	*MSMEG_0965*	400ins[1]CC/ Asn135fs[2]	Porin M1
	MSMEG_1367	C2894T/ Ala965Val	DNA-directed RNA polymerase subunit beta (RpoB)
	MSMEG_1380	12insC/ Glu5fs	Transcriptional regulator, TetR family
	MSMEG_3244	1148_1152del[3]GCCCTinsCC/ Gly383_Pro384del insAla	Hypothetical protein
Msm-R1-13	*MSMEG_0452*	G850A/ Ala284Thr	GntP family permease
	MSMEG_0675	G142A/ Asp48Asn	Cytochrome P450
	MSMEG_1698	T305C/ Ile102Thr	AbrB family transcriptional regulator
	MSMEG_1740	361delG/ Val121fs	SDR family NAD(P)-dependent oxidoreductase
	MSMEG_1915	G306T/ Ter102Tyrext*?	Mycothiol system anti-sigma-R factor
	MSMEG_3033	1066delTinsGTG/ Cys356fs	3-Dehydroquinate synthase
	MSMEG_3033	T1088C/ Leu363Pro	3-Dehydroquinate synthase
	MSMEG_3244	1148_1152delGCCCTinsCC/ Gly383_Pro384del insAla	Hypothetical protein
	MSMEG_3522	A451G/ Thr151Ala	Dopamine receptor D4
	MSMEG_4780	A668G/ Tyr223Cys	Cytochrome P450
	MSMEG_5630	393insG/ Tyr132fs	GntR family transcriptional regulator
	MSMEG_6757	T74C/ Leu25Pro	IclR family transcriptional regulator
Msm-R3-2	*MSMEG_0241*	C2747T/ Pro916Leu	Transmembrane transport protein MmpL11
	MSMEG_0465	281insA/ Asn94fs	Transcriptional regulator, TetR family
	MSMEG_2624	268insC/ Leu90fs	Ribosome maturation factor RimP
	MSMEG_3244	1148_1152delGCCCTinsCC/ Gly383_Pro384del insAla	Hypothetical protein
	MSMEG_3987	C79T/ Leu27Phe	Hypothetical protein
	MSMEG_5085	G154T/ Ala52Ser	Dihydropteroate synthase 2
Msm-R4-1	*MSMEG_0241*	C2747T/ Pro916Leu	Transmembrane transport protein MmpL11
	MSMEG_0370	G1981A/ Ala661Thr	ATP-binding protein

Msm-R4-1	*MSMEG_0465*	281insA/ Asn94fs	TetR/AcrR family transcriptional regulator
	MSMEG_2624	268insC/ Leu90fs	UPF0090 protein
	MSMEG_3033	T1088C/ Leu363Pro	3-Dehydroquinate synthase
	MSMEG_3244	1148_1152delGCCCTinsCC/ Gly383_Pro384del insAla	Hypothetical protein
	The promoter region upstream of *MSMEG_3868*		Hypothetical protein
	MSMEG_3987	C79T/ Leu27Phe	Hypothetical protein
	MSMEG_5085	G154T/ Ala52Ser	Dihydropteroate synthase 2
Msm-R4-7	*MSMEG_0694*	G242T/ Arg81Leu	IniB N-terminal domain-containing protein
	MSMEG_1399	G199A/ Asp67Asn	30S ribosomal protein S7
	MSMEG_1481	G581T/ Arg194Leu	Class I SAM-dependent methyltransferase
	MSMEG_1515	G891A/ Gly291Arg	ATP-binding protein
	MSMEG_1686	A967C/ Met326Leu	C40 family peptidase
	The promoter region upstream of *MSMEG_1791*		Protein Usfy
	MSMEG_1791	1insC/ Met1fs	Protein Usfy
	MSMEG_2587	C262A/ His88Asn	Type I methionine aminopeptidase
	MSMEG_3605	C1190T/ Ser397Leu	NAD(P)-dependent alcohl dehydrogenase
	MSMEG_5078	G494T/ Cys165Phe	Glucose-1-phosphate adenylyltransferase
	MSMEG_6555	G703A/ Asp235Asn	TetR/AcrR family transcriptional regulator
	MSMEG_6768	C1553T/ Ala518Val	NAD(P)/ FAD-dependent oxidoreductase
	MSMEG_6798	A730G/ Met244Val	LacI family DNA-binding transcriptional regulator
	The promoter region upstream of *MSMEG_6819*		PfkB family carbohydrate kinase

[1] ins, insertion; [2] fs, frameshift; [3] del, deletion.

Table S3. MICs of multi-drugs against knockout strains and gene-edited strains.

**Strains**	**MIC (µg/mL)/fold change**				
	**RIF**	**EMB**	**STR**	**GEN**	**AMK**
Wt	16/1	1/1	0.5/1	2/1	0.5/1
Δ1380	16/1	1/1	0.5/1	2/1	0.5/1
Δ0965	32/2	1/1	0.5/1	2/1	0.5/1
Δ1380Δ0965	32/2	1/1	0.5/1	2/1	0.5/1
1380^{12insC}	16/1	1/1	0.5/1	2/1	0.5/1
$0965^{400insCC}$	32/2	1/1	0.5/1	2/1	0.5/1
1380^{12insC}-$0965^{400insCC}$	32/2	1/1	0.5/1	2/1	0.5/1

Wt, wild type Msm strain; Δ1380, *MSMEG_1380* knockout Msm strain; Δ0965, *MSMEG_0965* knockout Msm strain; Δ1380Δ0965, *MSMEG_1380* and *MSMEG_0965* double knockout Msm strain; 1380^{12insC}, Msm strain with a 12insC insertion mutation in *MSMEG_1380*; $0965^{400insCC}$, Msm strain with a 400insCC insertion mutation in *MSMEG_0965*; 1380^{12insC}-$0965^{400insCC}$, Msm strain with a 12insC insertion in *MSMEG_1380* and a 400insCC insertion in *MSMEG_0965*. RIF, rifampicin; EMB, ethambutol; STR, streptomycin; GEN, gentamicin; AMK, amikacin. The experiment was repeated three times.

Table S4. MICs of multi-drugs against recombinant strains.

**Strains**	**MIC (μg/mL)/fold change**								
	**VAN**	**CLR**	**SDZ**	**SMX**	**RIF**	**EMB**	**STR**	**GEN**	**AMK**
Wt	8/1	2/1	2/1	1/1	16/1	1/1	0.5/1	2/1	0.5/1
Msm::1380	8/1	2/1	2/1	1/1	16/1	1/1	0.5/1	2/1	0.5/1
Δ1380::C1380	32/1	2/1	2/1	1/1	16/1	1/1	0.5/1	2/1	0.5/1
Msm::0965	8/1	2/1	2/1	1/1	16/1	1/1	0.5/1	2/1	0.5/1
Δ0965::C0965	32/1	16/8	8/4	16/16	32/2	1/1	0.5/1	2/1	0.5/1

Wt, wild type Msm strain; Msm::1380, *MSMEG_1380* overexpression Msm strain; Δ1380::C1380, *MSMEG_1380* complemented Δ1380 strain; Msm::0965, *MSMEG_0965* overexpression Msm strain; Δ0965::C0965, *MSMEG_0965* complemented Δ0965 strain. VAN, vancomycin; CLR, clarithromycin; SDZ, sulfadiazine; SMX, sulfamethoxazole; RIF, rifampicin; EMB, ethambutol; STR, streptomycin; GEN, gentamicin; AMK, amikacin. The experiment was repeated three times.

Table S5. Primers used in this study.

**Primers**	**Primer sequences (5'-3')**	**Purpose**
Msm-clpP1-U	cggtcgggatccagcgac	Used for *clpP1* sequencing in Msm
Msm-clpP1-D	caggccgccgaggacagc	
Msm-clpP2-U	cgtggtgtttgccgttct	Used for *clpP2* sequencing in Msm
Msm-clpP2-D	ctggttcaccgcacagga	
Msm-prcA-U	tacgcgagcgccaccacg	Used for *prcA* sequencing in Msm
Msm-prcA-D	ggcggagaggacgacgatct	
Msm-prcB-U	cattgccgacggtcgcgaca	Used for *prcB* sequencing in Msm
Msm-prcB-D	cgacgcggattcggctctca	
Msm-1380-seq-F	cgctaccttccccgaactcg	Used for *MSMEG_1380* sequencing in Msm
Msm-1380-seq-R	ggccgccactgcgttacgag	
Msm-0965-seq-F	ccgaaaggtcagtcagcaaa	Used for *MSMEG_0965* sequencing in Msm
Msm-0965-seq-R	gatgaacggtgagacgttcg	
Msm-pRH-1380-CZF	ggcgcgtcgttgtggtcacctctagagtcgacctgcaggtt	To amplify the *MSMEG_0965* gene with *hsp60* promoter from Msm for cloning into the vector pRH2502
Msm-pRH-1380-CZR	gcccgccctgcgccacctgatctagatggccaccatggtg	
Msm-pRH-p0965-CZF	tgcattcatccaccggtctctctagagtcgacctgcaggtt	To amplify the *MSMEG_0965* gene with its own promoter from Msm for cloning into the vector pRH2502
Msm-pRH-p0965-CZR	aaccctggaacatgaactgatctagatggccaccatggtg	
QmmpS5-F	cctgtagtggtgacggagaag	Used for qRT-PCR
QmmpS5-R	gcatgccttcgagatccatg	
QmmpL5-F	cttctcgcaggccaagatg	
QmmpL5-R	gatcgtcgagtgttcgatcg	
QsigA-F	cgagcttgttgatcacctcgaccat	
QsigA-R	ctcgacctcatccaggaaggaac	

Table S6. CrRNAs used in this study.

**crRNAs**	**Description**
aacgacgtcgcgcgccacgc	crRNA for disruption of *MSMEG_1380* in Msm
acgagcacaggcacctctca	crRNA for disruption of *MSMEG_0965* in Msm
tgcccccgagacgccggagc	crRNA targeting the 12insCC of *MSMEG_1380* in Msm
ggtcatcaccccgaacctgt	crRNA targeting the 400insCC of *MSMEG_0965* in Msm
